# Effects of ZnO nanoparticulate addition on the properties of PMNT ceramics

**DOI:** 10.1186/1556-276X-7-65

**Published:** 2012-01-05

**Authors:** Methee Promsawat, Anucha Watcharapasorn, Sukanda Jiansirisomboon

**Affiliations:** 1 Department of Physics and Materials Science, Faculty of Science, Chiang Mai University, Chiang Mai 50200, Thailand; 2 Materials Science Research Center, Faculty of Science, Chiang Mai University, Chiang Mai 50200, Thailand

**Keywords:** ceramics, X-ray diffraction, microstructure

## Abstract

This research was conducted in order to study the effect of ZnO nanoparticulate addition on the properties of 0.9 Pb(Mg_1/3_Nb_2/3_)O_3_-0.1PbTiO_3 _[PMNT] ceramics. The PMNT ceramics were prepared by a solid-state reaction. The ZnO nanoparticles were added into PMNT ceramics to form PMNT/*x*ZnO (*x *= 0, 0.05, 0.1, 0.5, and 1.0 wt.%). The PMNT/*x*ZnO ceramics were investigated in terms of phase, microstructure, and mechanical and electrical properties. It was found that the density and grain size of PMNT ceramics tended to increase with an increasing amount of ZnO content. Moreover, a transgranular fracture was observed for the samples containing ZnO, while pure PMNT ceramics showed only a intergranular fracture. An addition of only 0.05 wt.% of ZnO was also found to enhance the hardness and dielectric and ferroelectric properties of the PMNT ceramics.

## Background

The complex perovskite Pb(Mg_1/3_Nb_2/3_)O_3 _[PMN] compound has been extensively studied for uses in several applications due to its high dielectric constant and low sintering temperatures [1-5]. The maximum dielectric constant [*ε*_rmax_] of PMN increased when normal ferroelectric PbTiO_3 _[PT] was added. The temperature related to this maximum (*T*_max_) also shifted upward [6]. The *ε*_rmax _of PMN reached the highest value with the addition of only 10 mol% PT [7,8]. The 0.9PMN-0.1PT [PMNT] is thus known as one of the most popular ferroelectric compositions which show a high dielectric constant and a high electrostrictive strain for multilayer capacitor and electrostrictive actuator applications. Under an actual working environment, however, PMNT ceramics still have problems related to mechanical strength. Moreover, for applications in electronic devices, high values of strength, hardness, and fracture toughness are also required. It is well understood that improving the densification process can effectively enhance the mechanical strength of ceramics. In addition, decreasing the grain size could also enhance the hardness and fracture toughness of ceramics [9,10]. According to previous investigations, one simple novel method to improve mechanical characteristics of oxide ceramics was based on the nanocomposite concept [11].

ZnO is known to have semiconductive properties and is now used in some electronic devices. It was found to improve sensitivity in materials used for sensing devices. Apart from this, the role of ZnO as a sintering aid in the sintering process was previously observed in ferroelectric ceramics such as PZT and PZT-BLT [9,10]. Moreover, addition of a ZnO nanoparticulate into these material systems also enhanced the hardness and fracture toughness of the ceramics. In this study, the ZnO nanoparticulate was thus selected as an additive for PMNT ceramics to improve mechanical properties, while dielectric and ferroelectric properties of the ceramics were expected to be maintained. Effects of the ZnO concentration on the phase, microstructure, and mechanical and electrical properties of PMNT ceramics were investigated and discussed.

## Methods

The PMNT powder was prepared by the columbite method [12]. The columbite precursor (MgNb_2_O_6_) was prepared by mixing the stoichiometric amounts of MgO (99.9%, Fluka, Sigma-Aldrich, St. Louis, MO, USA) and Nb_2_O_5 _(99.9%, Aldrich, Sigma-Aldrich, St. Louis, MO, USA) in ethanol, followed by ball milling for 24 h using a ZrO_2 _grinding medium. The slurry was dried at 120°C, and the powder was calcined at 1,000°C for 4 h. The columbite precursor was then mixed and ball-milled with predetermined amounts of PbO and TiO_2 _(99.9%, Aldrich) powders and calcined at 850°C for 2 h. The calcined powders were added with ZnO nanoparticles (20 nm, 99.5%, Nanostructured & Amorphous Materials, Inc., Houston, TX, USA) to form PMNT/*x*ZnO powders where *x *= 0, 0.05, 0.1, 0.5, and 1 wt.%. The mixed powders were then uniaxially pressed into pellets and sintered at 1,150°C for 2 h in an atmosphere of PMN powder. Bulk density of the ceramics was determined using Archimedes' method. Phase composition of the PMNT/ZnO ceramics was characterized using an X-ray diffraction method [XRD] (X-pert, PANalytical B.V., Almelo, The Netherlands). Microstructure of the ceramics was observed via a scanning electron microscope [SEM] (JSM-6335F, JEOL Ltd., Akishima, Tokyo, Japan). Average grain size was determined using a mean linear interception method from the SEM micrographs. In this method, a number of straight lines were drawn on each micrograph, and intercepted lengths of grains were obtained and averaged. The well-polished ceramics were subjected to Vickers indentation (Galileo Microscan, LTF S.p.a., Antegnate, Italy) for hardness (*H*_V_) determination. Fracture toughness (*K*_IC_) was determined following the method described by Antis et al. [13]. Dielectric constant and loss tangent were measured using an LCR meter (Hitester 3532-50, Hioki, Ueda, Nagano, Japan). Ferroelectric hysteresis (P-E) loops were characterized using a computer-controlled modified Sawyer-Tower circuit.

## Results and discussion

Relative density values of the PMNT/ZnO ceramics were measured and tabulated in Table 1. The results indicated that an addition of ZnO did not significantly change the relative density value of PMNT ceramics. However, the highest relative density value was obtained for the PMNT ceramic incorporated with 0.05 wt.% ZnO. This result was expected that the small amount of ZnO addition more effectively affected the densification process of the ceramic. Further addition of ZnO could more effectively influence the grain growth process according to the increase in grain size of the ceramics as shown in Table 1.

**Table 1:** Relative density, grain size, and lattice parameter of PMNT/ZnO ceramics

ZnO content (wt.%)	Relative density (%)	Grain size (μm)	Lattice parameter (Å)
0	96.62	1.88 ± 0.05	4.0344
0.05	96.87	2.15 ± 0.06	4.0370
0.1	96.66	2.61 ± 0.08	4.0421
0.5	96.78	2.71 ± 0.06	4.0438
1.0	96.67	3.07 ± 0.07	4.0447

**Figure 1:**
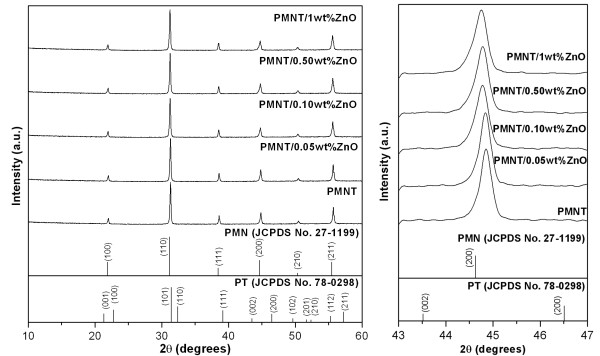
**XRD patterns of PMNT/ZnO ceramics sintered at 1,150°C and XRD peak at *2θ *≈ 45°**. The figure on the left showed XRD patterns of PMNT/*x*ZnO ceramics, where *x *= 0, 0.05, 0.1, 0.5, and 1 wt.% of CuO measured from an angle range of 10° to 60°. The other on the right showed a detail observation at angle ≈45° which indicated the shifting of a peak with ZnO additions.

Results of the phase characterization of PMNT/ZnO ceramics are shown in Figure 1. The XRD patterns were well matched with standard JCPDS file no. 27-1199 for the cubic phase in the $\text{Pm}\overset{-}{3}\text{m}$ space group. The XRD patterns showed that an addition of ZnO did not change the crystal structure of PMNT ceramics as well as no secondary phases including the ZnO phase were observed. The result suggested that Zn^2+ ^ions could completely enter into the lattice of the PMNT structure (within the limitations of the XRD technique). Moreover, a detailed observation of XRD peaks at *2θ *≈ 45° showed that the peaks were slightly shifted to the left with an increasing ZnO content. It was believed that the substitution of Zn^2+ ^ion (*r*_Zn2+ _= 0.74 Å) for Mg^2+ ^(*r*_Mg2+ _= 0.72 Å) or Nb^5+ ^ion (*r*_Nb5+ _= 0.64 Å) [14] in the B-site lattices of PMNT resulted in the expansion of the unit cell. This result was supported by the increasing values of the calculated lattice parameter as shown in Table 1.

**Figure 2:**
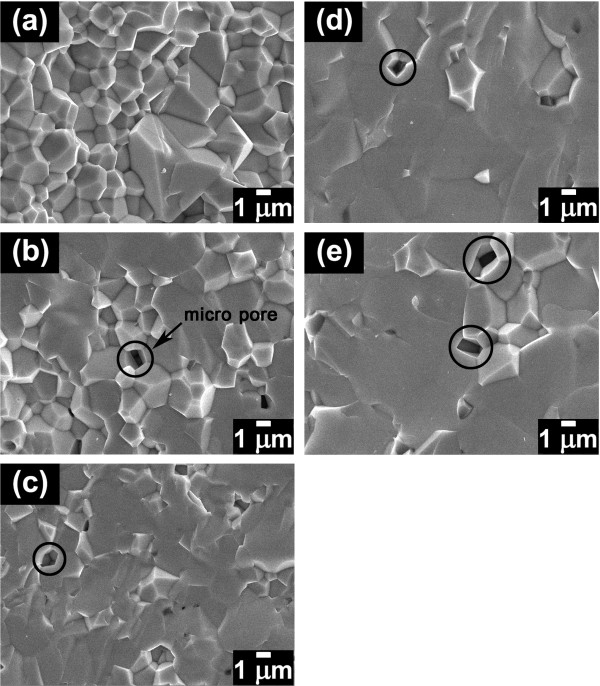
**SEM images of the fractured surface of PMNT/ZnO ceramics**. Fractured surface of pure PMNT ceramic (**a**) and PMNT ceramics incorporated with 0.05 (**b**), 0.1 (**c**), 0.5 (**d**), and 1 (**e**) wt.% ZnO.

SEM micrographs of the fractured surface of PMNT/ZnO ceramics are shown in Figure 2. Average grain sizes tabulated in Table 1 indicated that the grain size sharply increased when 0.05 to 0.1 wt.% ZnO was added. It was believed that this behavior was due to the enhancement of mass transport caused by ZnO addition [15] which led to more grain growth. However, the grain size was quite constant with further ZnO addition (0.5 to 1.0 wt.%). In this case, some undetected ZnO may partially distribute at grain boundary and act as a grain growth inhibitor. Microstructure of the pure PMNT as shown in Figure 2a revealed mainly an intergranular fracture. The samples incorporated with ZnO nanoparticles show a mixed-mode of inter/transgranular fracture as shown in Figure 2b, c, d. The degree of transgranular fracture tended to predominantly occur in large grains. This result indicated that large grains were weaker than smaller ones [16]. Moreover, the result may also be caused by the pinning at grain boundary of added ZnO contributing to crack deflection into grain bulk which caused a higher occurrence of transgranular fracture in large grains.

**Figure 3:**
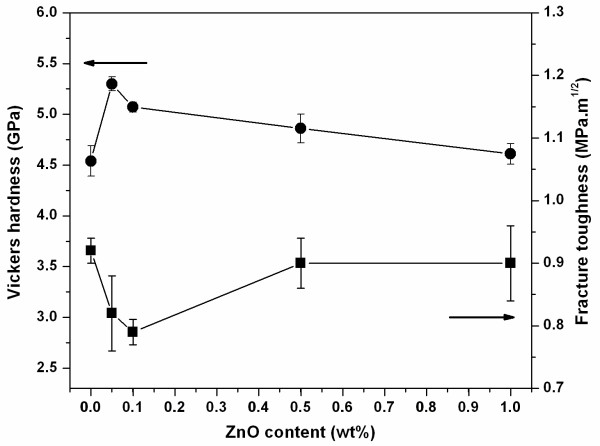
**Vickers hardness and fracture toughness of PMNT/ZnO ceramics**. The upper line indicated the relation of Vickers hardness and ZnO content. The other lower line indicated the relation of fracture toughness and ZnO content.

Mechanical properties of the ceramics in terms of Vickers hardness (*H*_V_) and fracture toughness (*K*_IC_) were investigated, and the results are shown in Figure 3. The hardness value of the pure PMNT ceramic was approximately 4.5 GPa, and the value increased to approximately 5.3 GPa when 0.05 wt.% of ZnO was added into the PMNT ceramic. The ZnO solute in the PMNT grain was believed to contribute to higher resistance to Vickers indentation, leading to a harder material. Among PMNT ceramics incorporated with ZnO, however, the hardness value slightly decreased with further increasing ZnO content. The decrease of hardness value was associated with an increase of grain size. It was known that grain boundaries in the ceramic having smaller grains are stress concentration sites which acted as effective obstacles to dislocation pile-up in the adjacent grains, leading to a harder material [17]. Fracture toughness result showed that an addition of 0.05 to 0.1 wt.% ZnO decreased fracture toughness values of PMNT ceramics. Due to the increase in grain size and observation of transgranular fracture when the amount of ZnO content was increased, the crack length of PMNT/ZnO ceramics extended longer than that in the pure PMNT ceramic, leading to a decrease in the fracture toughness. Further increasing ZnO content (0.5 to 1.0 wt.%) slightly increased the fracture toughness of the ceramics. It was believed that the micropores as observed at grain boundaries in the PMNT/ZnO ceramics (in black circles in Figure 2b, c, d) contributed to the obstruction of crack propagation, and hence, a decrease in crack propagation led to an increase in fracture toughness values.

**Figure 4:**
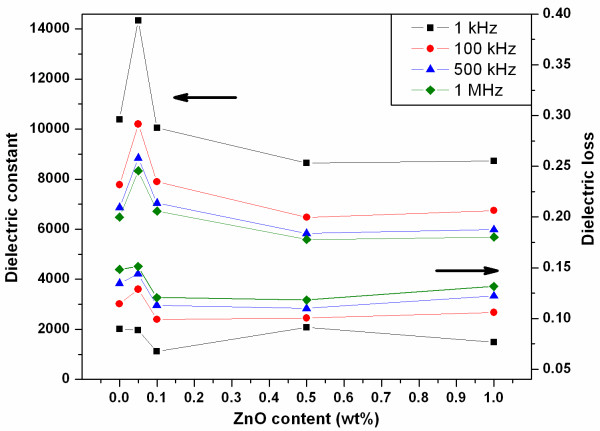
**Dielectric constant and dielectric loss of PMNT/ZnO ceramics measured at room temperature**. The upper group showed the relation of dielectric constant and ZnO content. The other lower group showed the relation of dielectric loss and ZnO content. These values were obtained from sample measurement at frequencies of 1 kHz (black line and square symbol), 100 kHz (red line and circle symbol), 500 kHz (blue line and triangle symbol), and 1 MHz (green line and tilted square symbol).

Dielectric constant and dielectric loss values measured at room temperature and plotted as a function of ZnO content are shown in Figure 4 and tabulated in Table 2. Typical characteristics of relaxor ferroelectrics, i.e., decreasing of dielectric constant and increasing of dielectric loss values with an increasing frequency, were observed in this ceramic system. An addition of 0.05 wt.% ZnO sharply increased the dielectric constant value of the ceramics. The dielectric constant values seemed to be correlated to the density values of PMNT/ZnO ceramics. From the above relationship, dielectric constant values of ceramics with further increasing of ZnO content (0.1 to 1.0 wt.%) were believed to be due to the presence of micropores at grain boundaries of the ceramics.

**Table 2:** Dielectric and ferroelectric properties of PMNT/ZnO ceramics

ZnO content (wt.%)	Dielectric properties^a^		Ferroelectric properties^a^		Loop squareness (*R*_sq_)
	*ε*_r_	tan *δ*	*P*_r_*/P*_max_	*E*_c_*/E*_max_	
0	10380	0.0896	0.37	0.13	0.49
0.05	14344	0.0887	0.29	0.10	0.44
0.1	10051	0.0677	0.32	0.11	0.41
0.5	8650	0.0913	0.32	0.10	0.42
1.0	8734	0.0769	0.38	0.12	0.47

^a^Dielectric and ferroelectric properties are measured at room temperature with a frequency of 1 kHz and 20 Hz, respectively.

**Figure 5:**
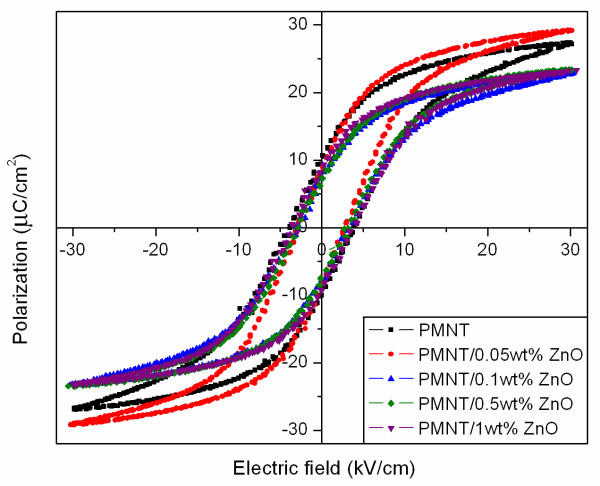
**Hysteresis loops of PMNT/ZnO ceramics measured at 20 Hz**. The figure showed the relation of the applied alternative current electric field at 20 Hz and measured polarization of the pure PMNT ceramic (black line and square symbol) and PMNT ceramics incorporated with 0.05 (red line and circle symbol), 0.1 (blue line and face up triangle symbol), 0.5 (green line and tilted square symbol), and 1 (violet line and face down triangle symbol) wt.% ZnO.

Hysteresis loops of PMNT/ZnO ceramics are shown in Figure 5, and the related values (i.e., *P*_r_, *E*_c _and *R*_sq_) were evaluated and listed in Table 1. Because of the temperature and field dependence of ferroelectric properties of ceramics, these parameters were normalized in the form of *P*_r_/*P*_max _and *E*_c_/*E*_max _values [18]. The hysteresis loop was well developed when 0.05 wt.% ZnO was added. However, the hysteresis loop was suppressed with 0.1 to 1.0 wt.% of ZnO additions. The results were associated with dielectric characteristics of the PMNT/ZnO ceramics. As mentioned above, since dielectric properties depended on the densification behavior of the ceramics, ferroelectric property behavior was thus believed to be attributed to the densification behavior of the ceramics as well. Therefore, the important factor mainly affected by the electrical properties of PMNT/ZnO ceramics in this study seemed to be the densification behavior of the ceramics.

## Conclusions

It can be seen that the PMNT/ZnO ceramics were successfully prepared by a solid-state mixed-oxide method. The variation of lattice parameters, microstructure, and mechanical and electrical properties of the ceramics were affected by the addition of ZnO nanoparticles. The hardness value of the pure PMNT ceramic increased from approximately 4.5 to approximately 5.3 GPa when 0.05 wt.% of ZnO was added into the ceramic. An addition of ZnO in the range of 0.5 to 1.0 wt.% tended to increase the fracture toughness value. Moreover, an addition of 0.05 wt.% ZnO enhanced the dielectric constant of the monolithic PMNT ceramic from 10,380 to 14,344. Furthermore, ferroelectric properties of the ceramic were also improved when 0.05 wt.% of ZnO was added. From this investigation, it was suggested that the optimum composition of the PMNT/ZnO system would be 0.05 wt.% ZnO due to its superior mechanical, dielectric, and ferroelectric properties.

## Competing interests

The authors declare that they have no competing interests.

## Authors' contributions

MP is the primary author who considered this study and carried out the experiments, characterization, acquiring of data, analysis of obtained data, and drafting of the manuscript. AW and SJ participated in the analysis and interpretation of the data and also in improving the language in the manuscript. All authors read and approved the final manuscript.
